# Skeletal Involvement of *Brucella melitensis* in Children: A Systematic Review

**Published:** 2013-12

**Authors:** Anahita Sanaei Dashti, Abdollah Karimi

**Affiliations:** 1 Shiraz HIV/AIDS Research Center, Shiraz University of Medical Sciences, Shiraz, Iran;; 2 Pediatric Infections Research Center, Mofid Children’s Hospital, Shahid Beheshti University of Medical Sciences, Tehran, Iran

**Keywords:** Brucellosis, Systematic review, Skeletal involvement, Arthritis, Sacroiliitis, Spondylitis, *Brucella melitensis*

## Abstract

Brucellosis is a protean disease and should be excluded in any febrile child with a constellation of symptoms such as fever, malaise, sweating, arthralgia, and joint swelling in endemic areas. Skeletal system involvement is the most common source of complaints in brucellosis. The frequency of skeletal involvement in children is 6.4% to 73.5%. There are some controversies regarding the most common sites of involvement: sacroiliac versus peripheral joints. In the vast majority of cases, peripheral joint involvement in pediatric brucellosis has a monoarticular pattern, although there is no agreement about the most commonly involved peripheral joint. In this systematic review, published articles that describe the bone involvement of *Brucella melitensis*, as the most prevalent kind of the microorganism in the region, in children are evaluated.

## Introduction

Brucellosis, previously known as Malta fever, is one of the most common zoonotic diseases. Owing to its subtle nature, difficult diagnosis, tendency to relapse, and potentially debilitating complications, brucellosis is a major health problem in the world. Annually, more than half a million people are infected globally. This erratic illness was noted in the Mediterranean region by Hippocrates in 450 B.C. and was described by the Romans 2000 years ago. 

Brucellosis is endemic in Iran. However, according to the data reported by the National Commission on Communicable Diseases Control, the incidence of brucellosis is in decline in Iran. In 1989, the annual incidence surpassed 1000 cases per million;^1^ and in 2003, the annual incidence plummeted to 238.6 cases per million.^2^ Be that as it may, it seems that human brucellosis is still a significant burden in Iran.^3^

Brucellosis is caused by organisms belonging to the genus *Brucella*, which is an aerobic and non-motile Gram-negative intracellular bacterium that does not produce spores. This genus comprises seven species based on antigenic and host differences: *B. melitensis* (sheep and goats); *B. abortus* (cattle); *B. suis* (pigs); *B. ovis* (sheep); *B. canis* (dogs); *B. neotomae* (rats); and *B. maris* (marine mammals).

Brucellosis is a febrile illness with a few vague systemic complaints, placing it in the differential diagnosis of many feverish diseases. Bone and joint involvements including arthritis, spondylitis, and osteomyelitis, are the most common complications of brucellosis. Kennedy made the first report of the skeletal involvement of brucellosis in 1904, almost 20 years after the discovery of the Malta fever bacterium by Sir David Bruce.^4^ There are several published reports of the skeletal involvement of brucellosis from different regions; nonetheless, a consensus has yet to emerge as to the prevalence, location, and type of involvement in children.

The purpose of the present study was to review and summarize the reports of the skeletal system involvement of *B. melitensis* in children.

## Methodology

*Inclusion Criteria*

Reviewed studies were of the observational type, exclusively in the age group of children, or studies that compared children and adults. As another requirement, the diagnosis of brucellosis had to be based on the presence of relevant clinical complaints associated with positive blood or bone marrow cultures or serology (positive Wright test result of 1/160 or more). Due to the high virulence of *B. melitensis*, its tendency to produce skeletal complications, and its prevalence in the Middle East region, we restricted the review to articles that considered *B. melitensis* as their sole or most frequent etiologic agent.

*Search Engines*

To find the eligible articles, we employed the search engines of Google Scholar, PubMed, and Cochrane database. The following journal sites were also directly investigated: 

(1) International Journal of Infectious Diseases 

(2) Lancet: The Infectious Diseases Collection 

(3) The Pediatric Infectious Disease Journal 

(4) Clinical Infectious Diseases Journal

*Keywords*

**Figure 1:**
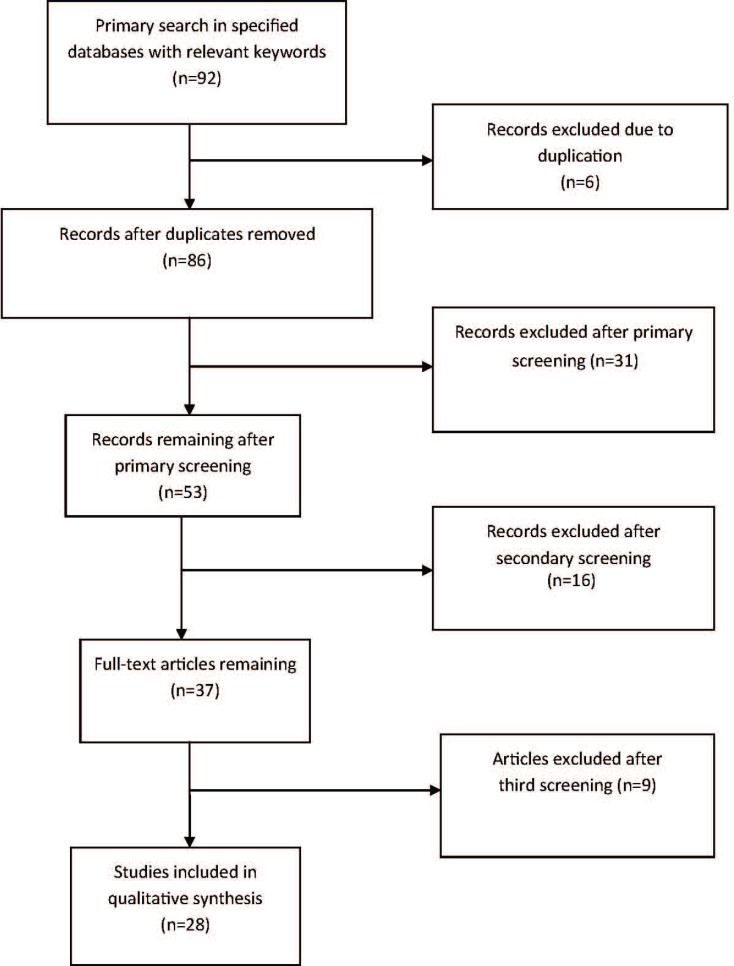
The Moose flowchart for article screening

Search was done via the keywords of brucellosis, *melitensis*, children, arthritis, osteomyelitis, spondylitis, skeletal manifestations, and sacroiliitis in English and Persian. Search was performed on two separate occasions by two separate researchers from January 2009 until March 2012 on data that were published after 1980 (figure 1).

## Results

*Prevalence of Skeletal Complications*

Prevalence of the skeletal complications of brucellosis in the published articles varied from 11 to 85%.^4^^-^^7^ The reported prevalence was influenced by factors such as *brucella* species, differences in hosts and populations, environmental factors, patient’s age, disease duration, and differences in the diagnostic methods and criteria.^4^^,^^6^ Complaints arising from the skeletal system were the main reason for seeking medical attention in children.^8^ In the Gür^5^ study on distinct groups of adults and children, the skeletal complications of brucellosis were more frequent in the children than in the adults (table 1).

**Table 1:** Frequency of the skeletal involvement of brucellosis in children and adults (in percentages

**Investigator**	**Children**	**Adults**
Gotuzzo^9^	13.6	26.7
Al Shamahy^10^	6.4	19.7
Gür^5^	73.5	68.5

*Location and Character of Skeletal Involvement*

It seems that the most prevalent location of the skeletal involvement of brucellosis in adults is the sacroiliac joint.^6^^,^^11^ However, some researchers believe that in brucellosis, peripheral joint involvement is more common than is sacroiliitis.^9^^,^^12^^,^^13^ There was no agreement between four studies that exclusively appraised the skeletal involvement of brucellosis in children with respect to the most frequent site of the involvement of the disease (tables 2 and 3). According to the studies of Gür^5^ and Geyik^6^ in children, sacroiliac and peripheral joints were equally affected in brucellosis, whereas Al-Eissa^4^ and Gómez^12^ reported that the involvement of peripheral joints was more common than that of the sacroiliac joint in children.

**Table 2:** Frequency of the skeletal involvement of brucellosis in children according to different studies (reported in percentages

**Investigator**	**Number of patients with brucellosis**	**Frequency of skeletal involvement**
Gómez–Reino^12^	36	30
Gür ^5^	53	73.5
Shaalan^10^	115	75
Almuneef^14^	62	19
Al–Eissa^4^	102	38
Mousa^15^	30	37.4
Gotuzzo^9^	22	13.6
Mantur^16^	93	38.7
Roushan^17^	35	37
Zamani^18^	96	25
Galanakis^19^	52	60

**Table 3:** Comparison of the sites of the skeletal involvement of brucellosis between children and adults

**Investigator**	**Type of involvement**					
		**Sacroiliitis**	**Peripheral ** **arthritis**	**Spondylitis**	**Osteomyelitis**	**Periarticular swelling**
Gür ^5^	Children	50	50	17	0	5
	Adults	62.2	56.5	24.5	8	4
Geyik^6^	Children	48.7	48	17.9	0	5.12
	Adults	62.2	56.5	24.5	24.5	4.09
Al-Eissa^4^	Children	8	93	0	5	NR
Gomez^12^	Children	4.8	95.2	0	0	NR

NR: Not reported

*Involvement of Peripheral Joints*

Both types of direct joint involvement (septic arthritis) and reactive arthritis may occur in brucellosis. Peripheral joint involvement, including knee, hip, ankle, shoulder, wrist, and elbow as well as even sternoclavicular joints, has been reported in brucellosis. Involvement of the small joints of hands and feet is rare. However, Shen^20^ reported the involvement of the proximal interphalangeal joints. Overall, probably the most common form of the skeletal involvement of brucellosis in children is peripheral arthritis. Arthritis can be acute (3 months), sub-acute (3 to 12 months), or chronic (more than 12 months).

As is shown in table 4, and according to various studies, peripheral joint involvement in children with brucellosis ranges from 13.6% to 50%. 

**Table 4:** Frequency of peripheral arthritis in children suffering from brucellosis

**Investigator**	**Number of cases**	**Location**	**Arthritis (per cent)**	**Monoarticular** **type (per cent)**
Shen^20^	20	Texas	50	80
Feiz^21^	95	Iran	19	often
Sahrda^22^	200	Kuwait	30	69
Mantur^16^	93	India	38.7	81
Al-Eissa^4^	102	Saudi Arabia	36	71
Al-Shamhy^23^	47	Yemen	6.4	NR
Shaalan^10^	115	Saudi Arabia	71	90
Gotuzzo^9^	22	Peru	13.6	75
Roushan^17^	111	Iran	31.5	82.8
Benjamin^24^	157	Saudi Arabia	50	67
Zamani^18^	96	Iran	25	62.5
Galanakis^19^	52	Greece	60	45

NR: Not reported

According to the Al-Eissa’s^4^ study on 40 children suffering from *Brucella* arthritis, pain, soft tissue swelling on the joint, limitation of motion, and warmth were almost always present and, occasionally, some degrees of erythema or joint effusion were observed. This fact was confirmed in other studies carried out on children.^8^^,^^12^^,^^20^^,^^5^ Interestingly, 36 out of the 40 studied children with *Brucella *arthritis had arthralgias in joints without arthritis. In a study on children by Gómez,^12^ 50% of the patients with *Brucella* arthritis had arthralgias in joints without arthritis. Arthralgias presented as intermittent or migratory pain in small or large joints (or both) with no movement restriction.^4^

There is no consensus about the most commonly involved peripheral joint in pediatric brucellosis (table 5). While some studies cited the hip and some the knee, Gomez^12^ reported the ankle as the most frequently involved peripheral joint. In the vast majority of the cases, peripheral joint involvement in pediatric brucellosis had a monoarticular pattern. Al-Eissa^4^ reported that two thirds of the joints studied were affected as the monoarticular and the remaining as the pauciarticular type. In the pauciarticular type of arthritis, involvement was more additive than migratory. Also, in studies by Geylik,^6^ Mantur,^16^ and Shen^20^ on children, between 80 to 90% of the joint involvements in brucellosis were of the monoarticular type.

**Table 5:** Most common sites of the involvement of peripheral arthritis in children with brucellosis

**Investigator**	**Involved joints**
Al-Eissa^4 ^	Hip<knee<other joints
Geyik^6 ^	Hip<knee<ankle<other joints
Mantur^16 ^	Hp<knee
Gomez^12^	Ankle<hip<knee
Shaalan^10 ^	Hip≈knee<ankle
Lubani^25 ^	Hip≈knee
Roushan^17 ^	Hip≈knee
Shen^20 ^	Knee<sacroiliac<hip
Zamani^18^	Knee<hip<ankle
Galanakis^19^	Hip<knee<ankle

**Sacroiliitis**

Sacroiliitis is commonly the dominant form of the skeletal involvement of brucellosis in adults and seems to be the most common form of skeletal involvement in the countries where *B. melitensis* is common.^6^^,^^8^ It is frequently reported from the Mediterranean and the Middle East regions, possibly due to a higher incidence of *B. melitensis* in these areas.^7^

The reported overall prevalence of sacroiliitis is controversial. In adults, the prevalence rates of zero (Al-Rawi^26^ [1989, Iraq, 17 patients]), 26% (Khateeb^11^ [1990, Kuwait]), and 45% (Colmenero,^27^ [1991]) have been reported. 

Sacroiliitis in its acute form generally produces severe pain and limitation of movement (standing/walking). Pain is usually felt as a vague discomfort in the lower back and buttocks. When the pain is not too severe, the patient is comfortable in the prone position, although the pain is felt when the patient turns from side to side, walks, or stands. In this instance, the patient’s problem may be confused with acute disc herniation or acute femoral fracture.^4^

Rajapakse^7^ argued that if the patient could slowly rotate his/her hip, it would be clinical evidence of the lack of involvement of the hip. If a moderate pressure on the sacrum of a patient lying in the prone position produces pain in the sacroiliac area, there is probably a pathology in that area. In such a case, a mild percussion on the heels of the patient lying in the supine position with extended hips may illicit pain in the sacroiliac region.^21^ Young^8^ highlighted the rarity of sacroiliac involvement in children. Geyik^6^ compared 39 children with 122 adults in terms of the skeletal involvement of brucellosis. According to the results, sacroiliitis constituted about 48.7% of all the skeletal involvement of brucellosis in the children compared to 62.2% in the adults. Sacroiliitis was unilateral in 84% of the pediatric cases and bilateral in the remaining. Bilateral sacroiliitis was generally significantly less frequent in the adults. Contrary to the high rate of sacroiliac involvement in the Geyik’s^6^ investigation, Al-Eissa^4^ reported sacroiliitis in 8% of the pediatric study population (n=40) with the skeletal involvement of brucellosis. This number was 5.5% in the Roushan^17^ study (table 6).

**Table 6:** Frequency of sacroiliac involvement (percentage of all skeletal involvement

**Investigator**	**Sacroiliitis**
Roushan^17^	5.5
Al-Eissa^4^	8
Geyik^6^	48.7
Gomez^12^	4.8

**Spondylitis**

Spondylitis possesses an insidious nature and produces mild pain despite the presence of obvious radiological signs.^28^ There is local tenderness or limitation of motion, or both. With an increase in the severity and extent of the disease, difficulty in walking and symptoms of pressure on the spinal cord may be reported on physical examination. The lumbar region is the most common site of involvement. Sometimes para-spinal abscesses also occur, though with a smaller size than that of tuberculous abscesses. An incidence rate of even up to 16% for these abscesses in brucellosis has been reported. 

Spondylolisthesis, paraplegia, and sphincter malfunction as a result of brucellosis have been reported. Unlike sacroiliac involvement, spinal involvement in brucellosis is often associated with graphic signs. Most of the time, brucellosis involves the anterior superior vertebral endplate, resulting in the epiphysitis of the anterior superior angle. Al-Eissa^4^ observed no case of brucellosis spondylitis in 40 children with skeletal complications. In a research by Geyik,^6^ 17.94% of the children and 24.59% of the adults suffering from the skeletal complications of brucellosis had spondylitis. In both age groups, the most common sites of involvement were the lumbar, dorsal, and cervical areas, respectively. In another study by Gür,^5^ very similar figures were obtained: 17% of the children and 24% of the adults with skeletal complications of brucellosis had spondylitis.

Some clinical and paraclinical findings of children and adults with brucellosis are compared in tables 7 and 8.

**Table 7:** Comparison of the frequency of paraclinical findings (reported in percentages) between children and adults with skeletal involvement of brucellosis

**Findings**	**Khateeb M (Adults)** ^11^	**Al-Eissa (Children)** ^4^
Anemia	13	40
Leukopenia	11	35
Lymphocytosis	40	NR
Leukocytosis	19	0
Neutrophilia	15	0
Neutropenia	NR	38
Elevated erythrocyte sedimentation rate (ESR)	39 (ESR>50)	43 (ESR>40)
Positive C-reactive protein	55	33
Positive antinuclear antibody	NR	25
Positive rheumatoid factor	3	21
Elevated liver enzymes	30	40
Thrombocytopenia	3	NR
Positive blood culture	22	75

NR: Not reported

**Table 8:** Comparison of the frequency of clinical findings (reported in percentages) between children and adults with skeletal involvement of brucellosis

**Complaint**	**Khateeb M (Adults)** ^11^	**Al-Eissa (Children)** ^4^
Arthralgia	100	90
Fever	93	93
Prostration	88	15
Low back pain	69	20
Myalgia	48	60
Anorexia	46	45
Weight loss	16	50
Splenomegaly (isolated)	21	25
Hepatomegaly (isolated)	6	20
Lymphadenopathy	NR	20

NR: Not reported

**Limitations**

Paucity of articles on the skeletal involvement of *B. melitensis* in children is the most important limitation of this review.

## Conclusion

Brucellosis is a protean disease and is reported to involve various areas of the skeletal system. Nevertheless, clarification of all the aspects of this issue in children requires a thorough and precise observation and documentation of the relevant data in the future. 
